# Dobutamine-sparing versus dobutamine-to-all strategy in cardiac surgery: a randomized noninferiority trial

**DOI:** 10.1186/s13613-021-00808-6

**Published:** 2021-01-26

**Authors:** Rafael Alves Franco, Juliano Pinheiro de Almeida, Giovanni Landoni, Thomas W. L. Scheeren, Filomena Regina Barbosa Gomes Galas, Julia Tizue Fukushima, Suely Zefferino, Pasquale Nardelli, Marilde de Albuquerque Piccioni, Elisandra Cristina Trevisan Calvo Arita, Clarice Hyesuk Lee Park, Ligia Cristina Camara Cunha, Gisele Queiroz de Oliveira, Isabela Bispo Santos da Silva Costa, Roberto Kalil Filho, Fabio Biscegli Jatene, Ludhmila Abrahão Hajjar

**Affiliations:** 1 Intensive Care Unit, Cancer Institute (ICESP), University of Sao Paulo, São Paulo, Brazil; 2 Department of Anesthesia and Intensive Care, IRCCS San Raffaele Scientific Institute, Milan, Italy; 3 Vita-Salute San Raffaele University, Milan, Italy; 4 Department of Anesthesiology, University Medical Center, Groningen, The Netherlands; 5 Department of Anesthesiology, Heart Institute, University of Sao Paulo, São Paulo, Brazil; 6 Division of Cardiovascular Surgery, Heart Institute (InCor), University of Sao Paulo, São Paulo, Brazil; 7 Department of Cardiology, Heart Institute (InCor), University of Sao Paulo, São Paulo, Brazil; 8 Department of Cardiopneumology, Instituto Do Coração (InCor), Hospital das Clínicas, Faculdade de Medicina da Universidade de São Paulo, Av. Dr. Enéas de Carvalho Aguiar, 44-05403-900, São Paulo, SP Brazil

**Keywords:** Cardiac surgery, Inotropes, Dobutamine, Inotrope sparing, Goal-directed therapy, Randomized clinical trial, Low cardiac output syndrome, Major cardiovascular events, Mortality

## Abstract

**Background:**

The detrimental effects of inotropes are well-known, and in many fields they are only used within a goal-directed therapy approach. Nevertheless, standard management in many centers includes administering inotropes to all patients undergoing cardiac surgery to prevent low cardiac output syndrome and its implications. Randomized evidence in favor of a patient-tailored, inotrope-sparing approach is still lacking. We designed a randomized controlled noninferiority trial in patients undergoing cardiac surgery with normal ejection fraction to assess whether an dobutamine-sparing strategy (in which the use of dobutamine was guided by hemodynamic evidence of low cardiac output associated with signs of inadequate tissue perfusion) was noninferior to an inotrope-to-all strategy (in which all patients received dobutamine).

**Results:**

A total of 160 patients were randomized to the dobutamine-sparing strategy (80 patients) or to the dobutamine-to-all approach (80 patients). The primary composite endpoint of 30-day mortality or occurrence of major cardiovascular complications (arrhythmias, acute myocardial infarction, low cardiac output syndrome and stroke or transient ischemic attack) occurred in 25/80 (31%) patients of the dobutamine-sparing group (*p* = 0.74) and 27/80 (34%) of the dobutamine-to-all group. There were no significant differences between groups regarding the incidence of acute kidney injury, prolonged mechanical ventilation, intensive care unit or hospital length of stay.

**Discussion:**

Although it is common practice in many centers to administer inotropes to all patients undergoing cardiac surgery, a dobutamine-sparing strategy did not result in an increase of mortality or occurrence of major cardiovascular events when compared to a dobutamine-to-all strategy. Further research is needed to assess if reducing the administration of inotropes can improve outcomes in cardiac surgery.

*Trial registration* ClinicalTrials.gov, NCT02361801. Registered Feb 2nd, 2015. https://clinicaltrials.gov/ct2/show/NCT02361801

## Background

Cardiac surgery has seen unanticipated progresses over the last decades, with dramatic reduction in postoperative mortality. However, patients are still at considerable risk for postoperative complications [1]. Low cardiac output syndrome is frequent after cardiac surgery, and a constant challenge for cardiac anesthesiologists and intensivists [2]. In an effort to avoid low cardiac output syndrome and its implications, standard management in many centers includes administering inotropes to all patients undergoing cardiac surgery [3–5]. A report from the Society of Thoracic Surgeons described how in the United States, more than 90% of patients undergoing coronary artery bypass graft surgery received vasoactive therapies [3]. The Contemporary Analysis of Perioperative Cardiovascular Surgical Care (CAPS-Care) study also reported how the percentage of patients receiving inotropic therapy was above 80% in over half of the participating centers, with many institutes administering inotropes as a standard of care in all patients undergoing cardiac surgery [4]. A recent survey conducted in Germany also showed a similar trend, reporting that three out of four cardiac surgery centers administer catecholamines to 80–100% of patients within the perioperative period [5]. No international guidelines suggest which inotrope should be the first-line agent in cardiac surgery, and local practices include a wide variety of strategies [6]. However, the Scandinavian Society of Anesthesiology and Intensive Care Medicine suggested that dobutamine should be used as the first-line agent in patients with shock after cardiac surgery [7]. While the detrimental effects of inotropic therapies are well-known, and a goal-directed therapy was effective in reducing complications after major noncardiac surgery [8, 9], randomized evidence supporting the use of goal-directed therapy in cardiac surgery is still lacking. The use of inotropic agents in cardiac surgery is controversial and was associated to an increased risk of major cardiovascular adverse events as ventricular arrhythmia, need for intra-aortic balloon pump and postoperative myocardial ischemia [10–12]. Even if the evidence suggesting that inotropes in cardiac surgery may be harmful only comes from low-quality observational studies, experts suggest to restrict their use only to those patients who have precardiotomy heart failure or have difficult separation from cardiopulmonary bypass (CPB) or postcardiotomy cardiogenic shock [13, 14].

Randomized evidence in favor of a patient-tailored, inotrope-sparing approach is still lacking. The aim of this study was to assess (whether an inotrope-sparing strategy, i.e., based on clinical and hemodynamic evidence of ongoing low cardiac output) is noninferior to an inotrope-to-all strategy in terms of clinically relevant outcomes after cardiac surgery.

## Methods

### Study design

Liberal Versus Restrictive Use of Dobutamine in Cardiac Surgery (DOBUTACS) study was a single-center, parallel randomized controlled noninferiority trial performed at the Heart Institute of the Sao Paulo University in Sao Paulo, Brazil, from February 2015 to April 2019. The protocol was approved by the ethics and research committee (Comissão de Ética para Análise de Projetos de Pesquisa—CAPPesq is 27534514.6.0000.0068, session of April 10, 2014) and registered at ClinicalTrials.gov (NCT02361801). The trial was overseen by an independent data and safety monitoring board. The study was sponsored by FAPESP (Fundação de Amparo a Pesquisa de São Paulo).

### Patients

Patients were screened for eligibility the day before surgery and written informed consent was obtained after a detailed explanation by the research staff. We included patients who were over 18 years old, scheduled for coronary artery bypass graft with CPB and with normal left ventricular ejection fraction (LVEF > 50%). Patients scheduled for combined or emergency surgery, those already receiving inotropes or with history of supraventricular or ventricular arrhythmias, pregnant women and patients already participating in other trials were excluded.

### Randomization and masking

Patients were randomly assigned to a dobutamine-to-all or to a dobutamine-sparing inotropic strategy in a 1:1 allocation ratio. A computer-generated list of random numbers was used to ensure allocation concealment. Participants were assigned to a progressive randomization number. The corresponding sealed, progressively numbered and opaque envelope containing information about patient allocation was opened by an independent trained researcher. The nature of the intervention precluded blinding of the attending physicians. Patients and outcome assessors were unaware of the assigned treatment. The outcome measures were assessed by consulting patient records. The vital status for patients already discharged from hospital was obtained through a telephone call.

### Trial interventions

At CPB separation, patients randomized to the dobutamine-to-all strategy received dobutamine, (starting dose: 5 mcg/kg/min). Patients allocated to the dobutamine-sparing strategy received dobutamine at CPB separation or postoperatively if they had evidence of low cardiac output, defined as a cardiac index of ≤ 2.4 L/min/m^2^. A conservative threshold of ≤ 2.4 L/min/m^2^ was used to recognize and treat low cardiac output syndrome before it became clinically overt [15, 16]. In both groups, dobutamine titration was based on clinical and hemodynamic condition of the patient. The anesthesiologist and the intensive care unit (ICU) physician increased or decreased dobutamine by 5 mcg/Kg/min every 30 min according to an institution protocol based on the presence of clinical signs of impaired perfusion, hemodynamic parameters, perfusion markers and oxygenation assessment. In both groups, dobutamine was weaned and discontinued as soon as the patient was clinically stable. All other interventions were at the discretion of the attending physicians. Fluids and norepinephrine were used upon clinical judgement of the attending anesthesiologist following an institutional protocol: fluid replacement was administered using dynamic parameters of fluid responsiveness; norepinephrine was started if mean arterial pressure was under 65 mmHg despite fluid resuscitation to treat vasoplegia.

Surgical and anesthetic procedures were performed according to the institutional protocol detailed in the Additional file 1: Material S1.

### Outcome measures, data collection and follow-up

The primary endpoint was a composite of 30-day mortality and major cardiovascular complications (ventricular or supraventricular arrhythmias, acute myocardial infarction, low cardiac output syndrome and stroke or transient ischemic attack).

Secondary endpoints were: incidence of CPB separation failure, need for mechanical circulatory support, need for additional inotropes, use of vasopressors and rate of acute kidney injury. We also registered changes in Sequential Organ Failure Assessment (SOFA) score [17] within 72 h after surgery, length of intensive care unit and hospital stay. During hospital stay we also collected data about the incidence of septic shock, rates of red blood cell transfusion, need for mechanical ventilation lasting longer than 48 h, need for renal replacement therapy, hemodynamic data and perfusion markers during ICU stay and ICU readmission rates. Outcome data were collected by a blind independent researcher daily at the bedside from the patients’ charts until hospital discharge and then stored in the appropriate case report form.

Comorbidities and endpoint definitions are reported in Additional file 1: Material 2.

### Sample size and data analysis

Sample-size calculation was based on a two-sided alpha error of 0.05 and an 80% power. On the basis of previous literature, we anticipated an incidence of 40% of the primary composite endpoint in the dobutamine-to-all group [18]. We identified a difference of 10% in the occurrence of the primary endpoint to be clinically important (− 10% was the margin used for the noninferiority design). Therefore, we anticipated that 160 patients (80 patients in each arm) would be needed to test our noninferiority hypothesis.

The noninferiority design was chosen as we believed that we could demonstrate even with a relatively small sample size that not using inotropes routinely may be a viable strategy in some patients, while larger studies are needed to affirm the superiority of a tailored inotrope administration strategy in cardiac surgery.

All analyses were conducted according to the intention-to-treat principle. No assumptions were made for missing or unavailable data. We reported continuous variables as mean and standard deviation (SD) or medians and interquartile range (IQR) and categorical variables as n (%). Continuous variables were compared using a Student’s *t* test or Mann–Whitney *U* test and categorical variables using Pearson Chi-square or Fisher exact or likelihood ratio test. A multiple logistic regression analysis was performed to identify independent predictors of the primary outcome. Comparisons of SOFA score over time were made using nonparametric Friedman test. Kaplan–Meier curves were built for event-free survival probability up to 30 days after surgery. A post hoc analysis was also performed to assess the occurrence of the primary outcome in patients receiving or not receiving preoperative beta-blockers. A test for interaction was run to assess the presence of statistically significant subgroup differences.

A two-sided *p*-value of less than 0.05 was considered statistically significant. Statistical analyses were performed using SPSS version 18.0 (SPSS, Chicago, IL).

## Results

### Study population

**Fig. 1:**
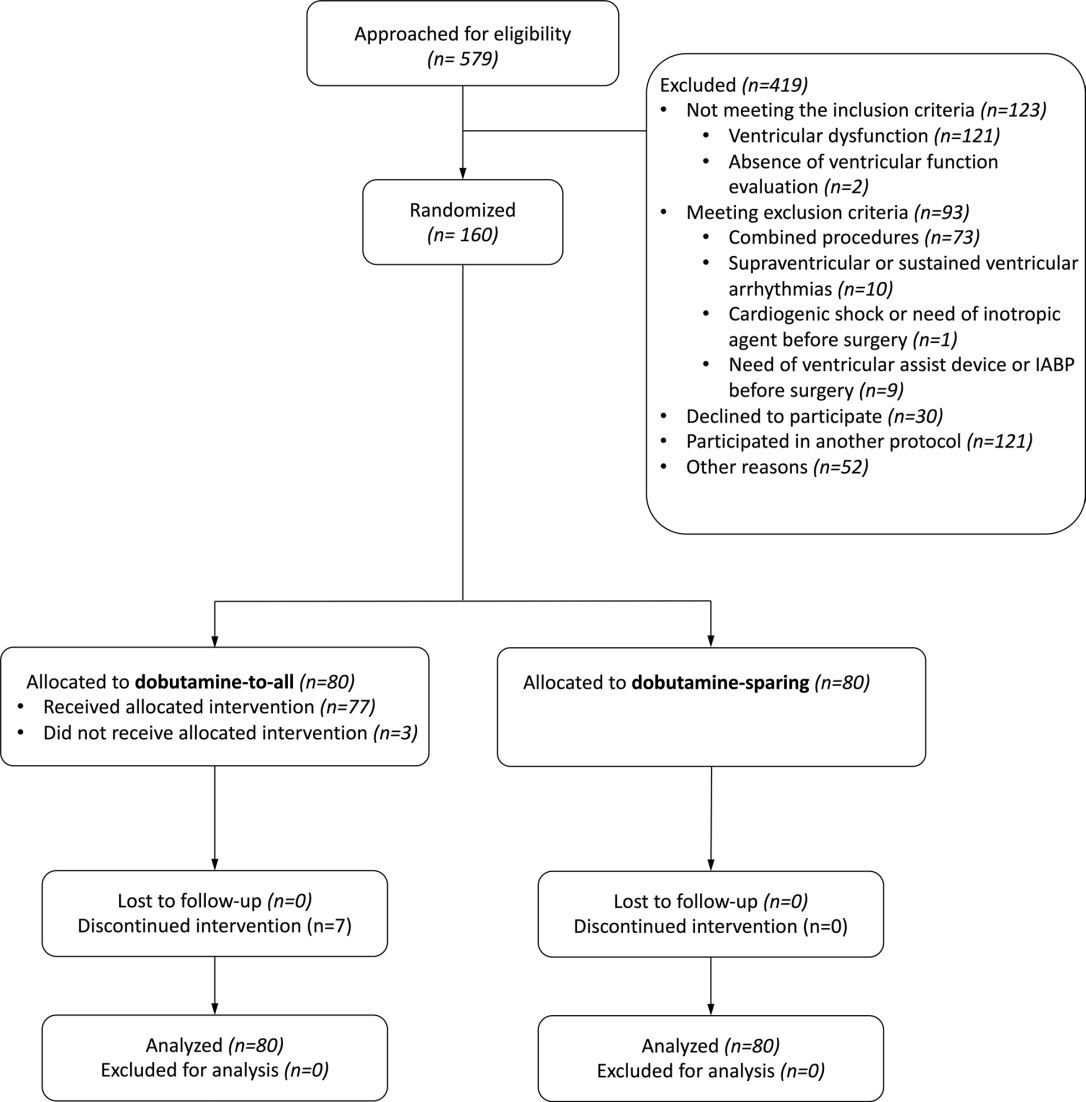
Study flowchart. IABP: intra-aortic balloon pump

One-hundred sixty patients were enrolled in the study: 80 patients were allocated to the dobutamine-sparing strategy, while 80 patients received the dobutamine-to-all approach (Fig. 1). There was no loss to follow-up during the study period and no patient withdrew consent.

The population of our study was 63 years old and 70% were male. The most common comorbidities were arterial hypertension, diabetes and dyslipidemia and the preoperative mean LVEF was 61%. Baseline characteristics of patients were similar between groups and are reported in Table 1. Intraoperative characteristics were well balanced between groups, including the percentage of patients receiving > 3 grafts and CPB duration. Intraoperative characteristics are described in Additional file 1: Table S1.

**Table 1:** Demographic data and preoperative characteristics according to the study groups: dobutamine-sparing (80 patients) and dobutamine-to-all (80 patients)

Variable	Dobutamine-sparing	Dobutamine-to-all	*p*-value
	*n* = 80	*n* = 80	
Age (years), median (IQR)	62 (55–68)	65 (57–69)	0.12^a^
Sex (male), *n* (%)	57 (71%)	59 (74%)	0.72^a^
BMI (kg/m^2^), median (IQR)	27 (25–31)	27 (24–30)	0.44^b^
Caucasian race, *n* (%)	71 (89%)	66 (83%)	0.26^a^
Smoking history, *n* (%)			
Current	15 (19%)	22 (28%)	0.19^b^
Previous (> 6 months)	28 (35%)	23 (29%)	0.43^b^
Right ventricular dysfunction, *n* (%)	2 (2.5%)	0	0.50^b^
Diastolic heart failure, *n* (%)	1 (1.3%)	0	0.99^d^
Acute myocardial infarction, *n* (%)	27 (34%)	25 (31%)	0.74^d^
Hypertension, *n* (%)	64 (80%)	69 (86%)	0.29^b^
Peripheral vascular disease, *n* (%)	3 (3.8%)	2 (2.5%)	0.99^b^
Chronic obstructive pulmonary disease, *n* (%)	1 (1.3%)	1 (1.3%)	0.99^d^
Dyslipidemia, *n* (%)	55 (69%)	50 (63%)	0.41^d^
Serum creatinine, median (IQR)	0.96 (0.77–1.28)	1.04 (0.83–1.26)	0.54^b^
History of atrial fibrillation, *n* (%)	0	2 (2.5%)	0.50^a^
Diabetes mellitus, *n* (%)	41 (51%)	33 (41%)	0.21^d^
Liver disease, *n* (%)	0	2 (2.5%)	0.50^b^
Hypothyroidism, *n* (%)	6 (7.5%)	4 (5.0%)	0.51^d^
Stroke, *n* (%)	1 (1.3%)	2 (2.5%)	0.99^d^
LVEF (%), median (IQR)	61 (57–68)	61 (56–66)	0.32^a^
ACE inhibitors and/or ARBs, *n* (%)	64 (80%)	68 (85%)	0.41^b^
Beta-blockers, *n* (%)	36 (45%)	32 (40%)	0.63^b^
Statin, *n* (%)	72 (90%)	70 (88%)	0.62^b^
EuroSCORE II, median (IQR)	2 (2–4)	2 (2–3)	0.10^a^
Charlson score, median (IQR)	3 (2–4)	3 (2–4)	0.43^a^
STS Score—mortality, median (IQR)	0.8 (0.6–1.1)	0.9 (0.7–1.3)	0.43^a^
STS Score—morbidity or mortality, median (IQR)	8.8 (8.0–12.0)	10.0 (7.4–11.7)	0.99^a^
Reoperation, *n* (%)	3 (3.8%)	5 (6.3%)	0.72^d^

*IQR* interquartile range, *BMI* body mass index, *LVEF* left ventricular ejection fraction, *ACE* angiotensin converting enzyme, *ARBs* angiotensin receptor blockers, *STS* Society of Thoracic Surgeons

^a^Mann–Whitney test

^b^Pearson's Chi-square test

^c^Likelihood ratio test; d: Fisher's exact test

### Intervention data, protocol deviations and adverse events

A total of 38/80 (48%) patients in the dobutamine-sparing group had a cardiac index ≤ 2.4 l/min/m^2^ and received dobutamine in the operating theater. Thirteen additional patients of the dobutamine-sparing group received dobutamine after ICU admission, while 29 patients (29/80, 36%) never received dobutamine (Additional file 1: Table S2).

In the dobutamine-to-all group all patients received dobutamine, with the exception of three patients in which the attending anesthesiologist deemed unsafe to initiate dobutamine due to clinical concerns about tachyarrhythmias. Four patients in the dobutamine-to-all group discontinued dobutamine administration in the ICU due to the occurrence of supraventricular arrhythmia which spontaneously converted to sinus rhythm shortly after stopping dobutamine infusion and which did not require any further therapy.

**Fig. 2:**
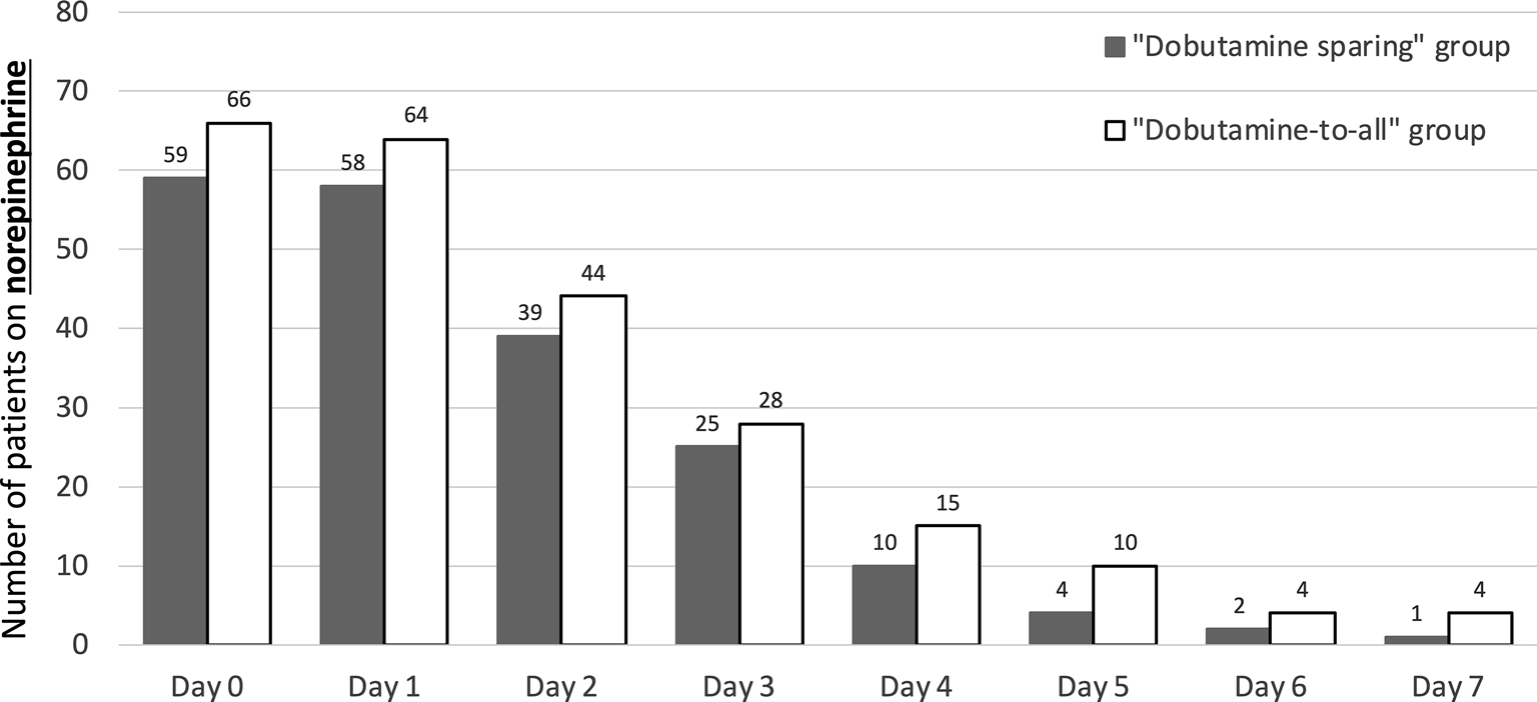
Use of norepinephrine in the first 7 days after surgery according to the study groups: dobutamine-to-all (80 patients) and dobutamine-sparing (80 patients)

**Fig. 3:**
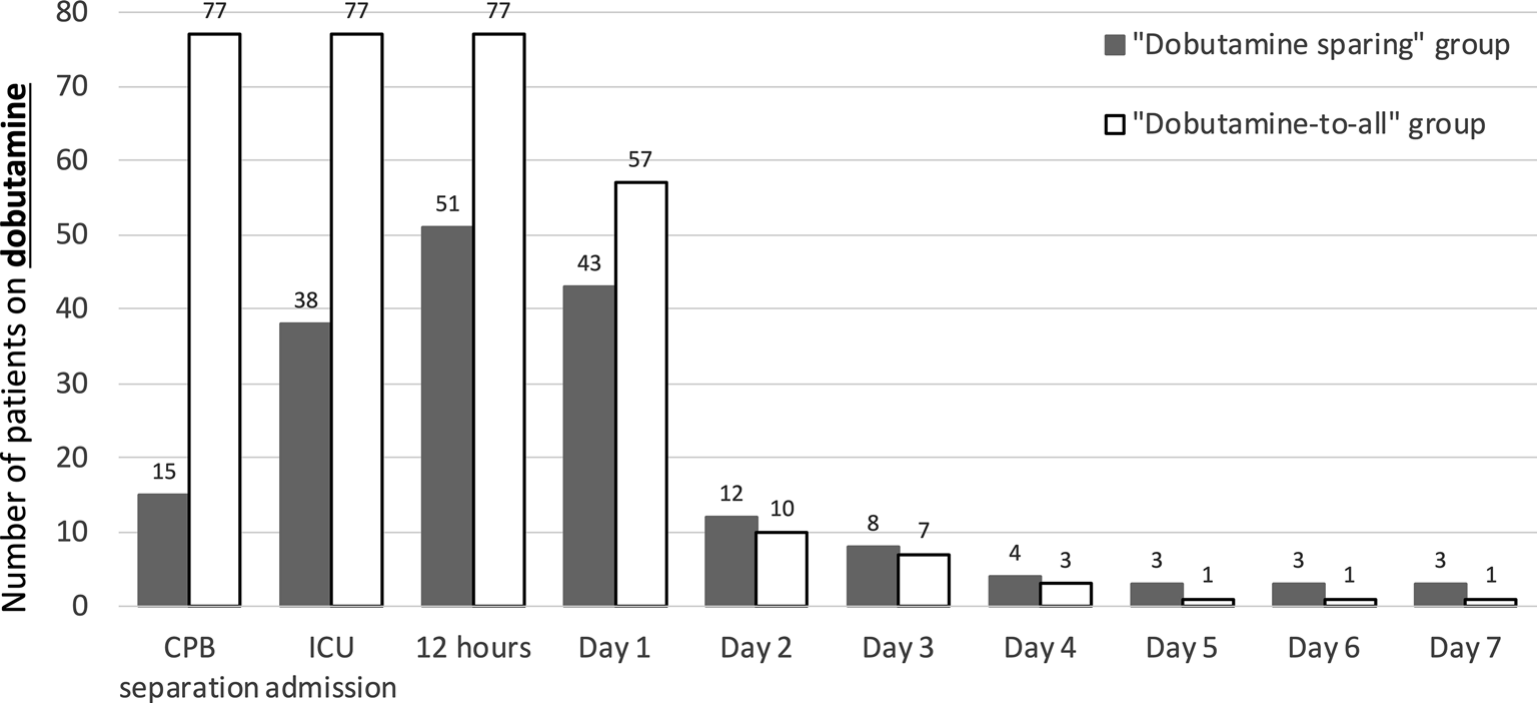
Dobutamine use during surgery and in the first 7 days postoperatively according to the study groups: dobutamine-to-all (80 patients) and dobutamine-sparing (80 patients). Note: Dobutamine was not started in 3 patients in the dobutamine group, as the attending anesthesiologist deemed it unsafe due to clinical concerns about tachyarrhythmias

Among patients who received inotropic therapy, the dose of dobutamine was similar between groups. The majority of patients in both groups received norepinephrine (Fig. 2). The number of patients receiving dobutamine over the study period is reported in Fig. 3, while dobutamine dosage is represented in Additional file 1: Figure S1.

### Outcomes

The primary composite outcome of 30-day mortality and major cardiovascular complications occurred in 27/80 (34%) in the dobutamine-sparing group versus 25/80 (31%) patients in the dobutamine-to-all group (*p* = 0.74, Table 2). There were no significant differences between the two groups in the incidence of supraventricular or ventricular tachyarrhythmia (24% vs 28%, *p* = 0.59), shock (14% vs 15%, *p* = 0.82), acute myocardial infarction (1.3% vs 2.5%, *p* = 0.99), stroke (2.5% vs 0, *p* = 0.50) and overall mortality (2.5% vs 6.3%, *p* = 0.44). The groups were also similar in the incidence of atrial fibrillation (24% vs 25%, *p* = 0.85), heart failure (14% vs 14%, *p* = 0.99), septic shock (3.8% vs. 1.3%, *p* = 0.62) and mechanical ventilation lasting longer than 48 h (3.8% vs 5.0%, *p* = 0–99). (Additional file 1: Figure S2)”.

**Table 2:** Primary and secondary outcomes according to the study groups: dobutamine-sparing (80 patients) and dobutamine-to-all (80 patients)

Variable	Dobutamine-sparing	Dobutamine-to-all	Absolute difference	*p*-value
	*n *= 80	*n* = 80	(95% CI)	
Primary composite endpoint	25 (31%)	27 (34%)	2.5 (− 11.8 to 16.7)	0.74^b^
Arrhythmias (supraventricular and/or ventricular), *n* (%)	19 (24%)	22 (28%)	3.7 (− 9.7 to 17.1)	0.59^b^
Low cardiac output syndrome^e^, *n* (%)	11 (14%)	12 (15%)	1.2 (− 9.9 to 12.4)	0.82^b^
Cardiogenic shock, *n* (%)	9 (11%)	11 (14%)	2.5 (− 8.1 to 13.1)	0.63^b^
Death, *n* (%)	2 (2.5%)	5 (6.3%)	3.8 (− 3.4 to 11.5)	0.44^d^
Acute myocardial infarction, *n* (%)	1 (1.3%)	2 (2.5%)	1.2 (− 4.5 to 7.5)	0.99^d^
Stroke, *n* (%)	2 (2.5%)	0	− 2.5 (− 8.7 to 2.4)	0.50^d^
Secondary outcomes				
Atrial fibrillation, *n* (%)	19 (24%)	20 (25%)	1.2 (− 12.0 to 14.4)	0.85^b^
Heart failure, *n* (%)	11 (14%)	11 (14%)	0 (− 10.9 to 10.9)	0.99^b^
Acute kidney failure (AKIN Stage ≥ 2), *n* (%)	10 (13%)	11 (14%)	1.3 (− 9.5 to 12.0)	0.82^b^
Renal replacement therapy, *n* (%)	7 (8.8%)	3 (3.8%)	− 5.0 (− 13.6 to 3.1)	0.19^b^
Septic shock, *n* (%)	3 (3.8%)	1 (1.3%)	− 2.5 (− 9.3 to 3.5)	0.62^d^
Mechanical ventilation > 48 h, *n* (%)	3 (3.8%)	4 (5.0%)	1.3 (− 6.1 to 8.8)	0.99^d^
Red blood cell transfusion, *n* (%)	10 (13%)	9 (11%)	− 1.2 (− 11.7 to 9.1)	0.81^b^
SOFA (72 h), median (IQR)	3 (2–6)	3 (1–5)	–	0.29^a^
Highest lactate (mmol/L), median (IQR)	3.9 (2.8–5.1)	4.0 (3.0–6.4)		0.47^a^
Length of ICU stay (days), median (IQR)	3 (2–5)	3 (2–4)	–	0.27^a^
Length of hospital stay (days), median (IQR)	14 (11–19)	13 (9–17)	–	0.25^a^
ICU readmission, *n* (%)	2 (2.6%)	1 (1.3%)	− 1.3 (− 7.5 to 4.5)	0.99^d^

*CI* confidence interval, *SOFA* Sequential Organ Failure Assessment, *IQR* interquartile range, *AKIN* Acute Kidney Injury Network

^a^Mann–Whitney test

^b^Pearson's Chi-square test

^c^Likelihood ratio test

^d^Fisher's exact test

^e^In spite of dobutamine administration

The SOFA score in the first 72 h was similar between the groups, with a median of 3 (2–6) in the dobutamine-sparing group and 3 (1–5) in the dobutamine-to-all group (*p* = 0.29) (Additional file 1: Figure S3). There was also no difference between groups regarding ICU length of stay [3 (2–5) vs 3 (2–4) days, *p* = 0.27] and hospital length of stay [14 (11–19) vs 13 (9–17) days, *p* = 0.25].

No differences between groups were observed in cardiac index, ScvO_2_, lactate levels, base excess and mean arterial pressure at different time points. (Additional file 1: Figure S4). The two groups also presented similar creatinine mean values (Additional file 1: Figure S5).

An exploratory subanalysis was performed to assess the incidence of the primary outcome in patients on preoperative beta-blockers and in patients not on preoperative beta-blockers. Primary outcome occurred in 13/32 (41%) patients on preoperative beta-blockers in the dobutamine-to-all group, versus 8/36 (22%) in the dobutamine-sparing group and in 14/48 (29%) patients not on preoperative beta-blockers in the dobutamine-to-all group, versus 17/44 (39%) in the dobutamine-sparing group (*p* for interaction = 0.063) (Additional file 1: Table S3).

## Discussion

In the present study, a dobutamine-sparing strategy was noninferior to a dobutamine-to-all strategy in patients undergoing coronary artery bypass graft with CPB in terms of 30 days mortality or major cardiovascular complications. In addition, there were no differences in the incidence of acute kidney injury, prolonged mechanical ventilation, ICU or hospital length of stay. The present randomized trial adds high-quality evidence on the effect of inotropes use on clinically relevant outcomes and might be of help in drafting future guidelines.

The controversy about using inotropic agents routinely in cardiac surgery patients was addressed multiple times in previous literature, but mostly through low-quality studies. Shahin et al. [19] analyzed a retrospective cohort composed of 1326 patients undergoing cardiac surgery and suggested that exposure to inotropic agents in the perioperative period of cardiac surgery was associated with an increased in-hospital mortality and renal dysfunction. Nielsen et al. [10], evaluating a prospective cohort of 6005 patients undergoing cardiac surgery, described an independent association between the use of dobutamine during the perioperative period of cardiac surgery and 30-day and 1-year mortality, as well as an increase in the incidence of acute myocardial infarction, stroke, arrhythmias, and renal replacement therapy. A recent manuscript describing over 100,000 patients undergoing cardiac surgery in 294 hospitals in the United States, reported that over 90% of patients received inotropes during hospitalization [20]. It is important to note, however, that most literature in this field is composed by nonrandomized trials, and no international guideline states that avoiding inotropes may be beneficial in cardiac surgery.

In the lack of strong evidence and guidelines, local practices widely differ around the world in terms of timing, type of inotrope and percentage of patients receiving inotropes. In many centers, both in the United States and in Europe, virtually all patients undergoing cardiac surgery receive inotropes [4–7, 20]. The results of our randomized clinical trial suggest that the routine use of dobutamine is not beneficial to patients, and might be detrimental. In fact, although not statistically significative, a 2.5-fold raise in mortality was observed in the dobutamine-to-all group (5/80 (6.3%) vs 2/80 (2.5%) which is worth further investigation in future trials. In the present study, a relevant number of patients assigned to the dobutamine-sparing strategy received dobutamine. Figure 3 shows how the use of dobutamine in the dobutamine-sparing group grew from 18% at CPB separation to 64% 12 h after surgery and then rapidly decreased, documenting that, even if 64% of patients were exposed to dobutamine in the restrictive strategy group, they received it for a short amount of time. It is possible that using more restrictive criteria to start dobutamine might have reduced the number of patients receiving dobutamine, possibly increasing the magnitude of our findings.

Our study has some limitations and several strengths. The trial was a single-center study performed in a national referral hospital in Brazil, which may compromise the generalizability of our data. The fact that not all perioperative physicians were blinded to the intervention may increase the risk of bias in the present study. However, due to the nature of the intervention, blinding the attending anesthesiologist would have put the patients at risk of life and major complications in a delicate setting such as the perioperative period of cardiac surgery and would have been therefore unethical. Also, our study reports an overall mortality of 4.4%, which some may consider high. Nevertheless, this data is in line with other randomized trials [21, 22] and substantially lower than what was reported from other Brazilian centers in previous experiences [23–25].

Criteria to start dobutamine were clearly stated in the protocol, and even if dosage titration was left to the judgement of the attending physician, we followed a precise institutional protocol. This is part of our pragmatic approach. Leaving to the attending physician discretion the inotrope dosage allowed for the trial to be performed without safety concerns for the enrolled patients. The present study adds randomized evidence to the use of different inotropic strategies in cardiac surgery (a field which is usually dominated by retrospective and observational studies), reporting complete mortality data and is therefore relevant and may play a role in the drafting of future guidelines in the field of cardiac surgery.

## Conclusions

In the present study, the adoption of a dobutamine-sparing inotropic strategy in cardiac surgery did not increase the incidence of mortality or major cardiovascular events when compared to a conventional approach in which dobutamine was administered to all patients. While international clinical guidelines on inotropes administration in cardiac surgery are still lacking, the present study suggests that “less is just as good” when it comes to catecholamines use in this setting.

## Supplementary Information

**Additional file 1: Material S1**. Institutional Protocol for surgical and anesthetic management in cardiac surgery. **Material S2.** Comorbidities and outcomes definitions. **Table S1.** Intraoperative characteristics of patients according to dobutamine group. **Table S2. **Dobutamine use during surgery and in the first 7 days postoperatively according to dobutamine group. **Table S3. **Primary outcome subanalysis according to preoperative beta-blockers therapy. **Figure S1**. Dobutamine dosage over time in the two group. **Figure S2**. Mechanical ventilation for the first 7 days postoperatively according to dobutamine group. **Figure S3.** SOFA score (mean and standard deviation) the first 7 days postoperatively according to dobutamine group. **Figure S4**. Hemodynamic variables (mean and standard deviation) during the first 7 days postoperatively according to dobutamine group. **Figure S5**. Creatinine during the first 7 days postoperatively according to dobutamine group. **Figure S6**. Hemoglobin during the first 24 hours according to dobutamine group.

## Data Availability

The datasets used during the current study are available from the corresponding author on reasonable request.

## Abbreviations

LVEF: Left ventricular ejection fraction
CPB: Cardiopulmonary bypass
ICU: Intensive care unit
SOFA: Sequential Organ Failure Assessment
SD: Standard deviation
IQR: Interquartile range
