# Supplementary material for: Dobutamine-sparing versus dobutamine-to-all strategy in cardiac surgery: a randomized noninferiority trial
Source: Ann Intensive Care. 2021 Jan 26;11:15. doi: 10.1186/s13613-021-00808-6 (PMC7838231; doi:10.1186/s13613-021-00808-6)

Additional file to

**Dobutamine-sparing versus dobutamine-to-all strategy in cardiac surgery: a randomized noninferiority trial**

*Rafael Alves Franco, Juliano Pinheiro de Almeida, Giovanni Landoni, Thomas W.L. Scheeren, Filomena Regina Barbosa Gomes Galas, Julia Tizue Fukushima, Suely Zefferino, Pasquale Nardelli, Marilde de Albuquerque Piccioni, Elisandra Cristina Trevisan Calvo Arita, Clarice Hyesuk Lee Park, Ligia Cristina Camara Cunha, Gisele Queiroz de Oliveira, Isabela Bispo Santos da Silva Costa, Roberto Kalil Filho, Fabio Biscegli Jatene, Ludhmila Abrahão Hajjar*

**Table of contents**

**Additional file 1: Material S1**. Institutional Protocol for surgical and anesthetic management in cardiac surgery Page 2

**Additional file 1: Material S2.** Comorbidities and outcomes definitions Page 4

**Additional file 1: Table S1.** Intraoperative characteristics of patients according to dobutamine group. Page 6

**Additional file 1: Table S2.** Dobutamine use during surgery and in the first 7 days postoperatively according to dobutamine group Page 7

**Additional file 1: Table S3.** Primary outcome subanalysis according to preoperative beta-blockers therapy Page 8

**Additional file 1: Figure S1**. Dobutamine dosage over time in the two groups Page 10

**Additional file 1: Figure S2**. Mechanical ventilation for the first 7 days postoperatively according to dobutamine group Page 11

**Additional file 1: Figure S3.** SOFA score (mean and standard deviation) the first 7 days postoperatively according to dobutamine group Page 12

**Additional file 1: Figure S4**. Hemodynamic variables (mean and standard deviation) during the first 7 days postoperatively according to dobutamine group. Page 13

**Additional file 1: Figure S5**. Creatinine during the first 7 days postoperatively according to dobutamine group. Page 14

**Additional file 1: Figure S6**. Hemoglobin during the first 24 hours according to dobutamine group Page 15

**Additional file 1: Material S1**. Institutional Protocol for surgical and anesthetic management in cardiac surgery

In the operating room, all patients had a central venous catheter and an arterial line, with a pulse contour cardiac output analysis using FloTrac/Vigileo monitor (FloTrac/Vigileo, Edwards Lifesciences, Irvine, California, USA). In both groups, patients received oral midazolam (0.1 to 0.2mg/kg) 30 minutes before surgery. Anesthesia was induced with fentanyl (3-5 μg/kg), midazolam (0.05mg/kg), etomidate (0.2-0.3mg/kg) and cisatracurium (0.2 mg/kg). Anesthesia was maintained with isoflurane in oxygen and fentanyl as needed. During surgery, additional doses of midazolam and cisatracurium were administered as required. After endotracheal intubation, Cefuroxime (1.500 mg) was administered intravenously at induction of anesthesia. Mechanical ventilation was set to intermittent positive pressure mode with a tidal volume of 6 mL/kg, positive end-expiratory pressure (PEEP) of 5 to 8 cm H_2_O, and fraction of inspired oxygen of 0.6 to 1 to keep the arterial oxygen saturation greater than 95%.

Blood glucose levels were kept between 140 and 180 mg/dL, using a continuous insulin infusion if needed. All surgical procedures were undertaken through a median sternotomy.

Anticoagulation started before initiation of CPB with an initial dose of 500 IU/kg of heparin to achieve a target activated clotting time of 480 seconds. At the end of CPB, heparin was reversed with protamine chloride in a 1:1 ratio, with additional doses as required to return the activated clotting time to preoperative values. Significant bleeding associated with platelet disorders or coagulopathy was treated using platelets, fresh frozen plasma, prothrombin complex concentrates, or cryoprecipitate.

A centrifugal pump (Medtronic Biomedicus; Medtronic, Minneapolis, Minnesota) was used for bypass. An extracorporeal circuit containing a microporous polypropylene membrane oxygenator (Braile; São José do Rio Preto, São Paulo, Brazil) with an integrated venous cardiotomy reservoir was used. The oxygenator was primed with 1500 mL of lactated *Ringer's* solution, 20% mannitol (1 g/kg), and 2500 units of unfractionated heparin. During bypass, a mild hypothermic temperature management strategy (32°C to 34°C) with α-stat blood gas management was used in all patients. During bypass, nonpulsatile flow was maintained at 2.0 to 2.4 L/min per square meter, and mean arterial pressure was kept from 60 to 90 mm Hg. Myocardial preservation was performed by intermittent antegrade cold crystalloid or blood cardioplegia during aortic cross-clamping. Lactated Ringer’s solution was infused according to filling pressures, diuresis, and cardiac output. Synthetic colloids were not used during the study. After the procedure, all patients were transferred to the surgical intensive care unit (ICU).

At the Heart Institute, all patients undergoing cardiac surgery are admitted in the Surgical ICU under mechanical ventilation. Extubation was accomplished after complete awakening from anesthesia, hemodynamic stability with no evidence of significant bleeding, core temperature equal or higher than 36^o^C, and adequate blood gas parameters. After ICU admission, a standardized protocol of care was implemented in all patients that aimed to reach the following goals: glycemia of 140-180 mg/dL, temperature of 36ºC, hematocrit level higher than 28%, electrolyte monitoring and replacement, hourly evaluation of analgesia and adequate control of agitation.

**Additional file 1: Material S2.** Outcomes definitions

**Myocardial infarction** was arbitrarily defined as elevation of cTn values >10 times the 99^th^ percentile upper reference limit in patients with normal baseline cTn values. In patients with elevated preprocedure cTn in whom cTn levels are sTable S(≤20% variation) or falling, the postprocedure cTn must rise by >20%. However, the absolute post-procedural value still must be >10 times the 99th percentile URL. In addition, one of the following elements is required:

- Development of new pathological Q waves;

- Angiographic documented new graft occlusion or new native coronary artery occlusion;

- Imaging evidence of new loss of viable myocardium or new regional wall motion abnormality in a pattern consistent with an ischemic aetiology.^1^

**Stroke or transient ischemic attack**: stroke was defined by a new focal deficit lasting for more than 24 hours and associated with a compatible image on brain computed tomography. Transient ischemic attack was defined by a new focal deficit lasting for less than 24 hours with a brain computed tomography without acute abnormalities.^2^

**Ventricular or supraventricular arrhythmias** were defined as tachyarrhythmias recorded by continuous telemetry lasting at least 30 seconds.^3^

**Low cardiac output syndrome** was defined as cardiac index of ≤2.4 L/min/m2 plus at least one clinical sign of low cardiac output urine output under 0.5 mL/kg/h for more than 1 hour, capillary refill time >3 seconds, bradycardia (heart rate inferior to 50 beats per minute) or altered mental status.

**Capillary refill time** was measured by squeezing the distal phalanx of the middle finger for five seconds. The finger was released, and a digital stopwatch was started. The watch was stopped when the finger pulp returned to its baseline color. Times were measured in 100ths of a second and rounded and recorded to the nearest tenth. The test was repeated on the index finger, and the two measurements were averaged.^4^

**Acute kidney failure** was defined according to the Acute Kidney Injury Network (AKIN) definition of Stage 2 or above Acute Kidney Injury: an abrupt (within 48 h) reduction in kidney function defined as a percentage increase in serum creatinine over 2-fold from baseline or a reduction in urine output (documented oliguria of < 0.5 mL/kg/h for >12 h).^5^

**Peripheral vascular disease** was assessed through an anamnestic report that included symptoms of peripheral arterial disease like intermittent claudication or muscle pain during exercises or walk, skin ulcers, hair loss on feet or legs, leg weakness, brittle toenails, numbness in the legs, and/or positive anamnesis for major vascular surgery.^6^

Additional file 1: References:

1. Thygesen K, Alpert JS, Jaffe AS, et al. Fourth universal definition of myocardial infarction (2018). *Eur Heart J* 2019; 40:237–269.

2. Gottesman RF, McKhann GM, Hogue CW. Neurological Complications of Cardiac Surgery. *Semin Neurol* 2008; 28:703–715.

3. Imazio M, Brucato A, Ferrazzi P, et al. Colchicine for Prevention of Postpericardiotomy Syndrome and Postoperative Atrial Fibrillation. The COPPS-2 Randomized Clinical Trial. *JAMA* 2014; 312:1016-1023.

4. Schriger DL, Baraff LJ. Capillary refill--is it a useful predictor of hypovolemic states?. *Ann Emerg Med* 1991; 20:601-605.

5. Mehta RL, Kellum JA, Shah SV, et al. Acute Kidney Injury Network: report of an initiative to improve outcomes in acute kidney injury. *Crit Care* 2007; 11:R31.

6. Steinmetz J, Rasmussen LS. Peri-operative cognitive dysfunction and protection. *Anaesthesia* 2016; 71 (Suppl. 1), 58–63

**Additional file 1: Table S1.** Intraoperative characteristics of patients according to dobutamine group.

| Variable | **Dobutamine-**  **sparing** | **Dobutamine-**  **to-all** | p-value | | | | |
| --- | --- | --- | --- | --- | --- | --- | --- |
|  | n=80 | n=80 |  |  |  |  |  |
| Total number of grafts, n (%) |  |  | 0.74 | ^a^ | |  |  |
| 1 | 2 (2.5%) | 1 (1.3%) |  |  | |  |  |
| 2 | 23 (29%) | 23 (29%) |  |  | |  |  |
| 3 | 40 (50%) | 45 (56%) |  |  | |  |  |
| >3 | 15 (19%) | 11 (14%) |  |  | |  |  |
| Grafts, n (%) |  |  |  |  | |  |  |
| Mammary | 78 (98%) | 79 (99%) | 0.99 | ^d^ | |  |  |
| Saphenous vein | 74 (93%) | 72 (90%) | 0.58 | ^b^ | |  |  |
| Radial | 4 (5.0%) | 2 (2.5%) | 0.68 | ^d^ | |  |  |
| CPB time (min), median (IQR) | 84 (72 - 109) | 92 (67 - 105) | 0.99 | ^a^ | |  |  |
| Aortic clamping time (min), median (IQR) | 64 (53 - 83) | 71 (49 - 84) | 0.47 | ^a^ | |  |  |
| Crystalloid (mL), median (IQR) | 2000  (1500 - 2500) | 2000  (1500 - 2500) | 0.32 | ^a^ | |  |  |
| Albumin supplementation, n (%) | 2 (2.5%) | 2 (2.5%) | 0.99 | ^d^ | |  |  |
| Water balance (mL), median (IQR) | +2525  (1825 - 3157) | +2855  (2337 - 3607) | 0.02 | ^a^ | |  |  |
| Red blood cell transfusion, n (%) | 15 (19%) | 18 (23%) | 0.56 | ^b^ | |  |  |
| Red blood cell concentrate, n (%) |  |  | 0.60 | ^c^ | |  |  |
| 0 | 66 (83%) | 62 (78%) |  |  | |  |  |
| 1 | 8 (10%) | 9 (11%) |  |  | |  |  |
| 2 | 6 (7.5%) | 8 (10%) |  |  | |  |  |
| 3 | 0 | 1 (1.3%) |  |  | |  |  |
| Fresh frozen plasma, n (%) | 1 (1.3%) | 0 | 0.99 | ^d^ | |  |  |
| Platelet concentrate, n (%) | 1 (1.3%) | 1 (1.3%) | 0.99 | ^d^ | |  |  |
| Vasopressor, n (%) | 27 (36%) | 29 (40%) | 0.60 | ^b^ | |  |  |
| Vasodilator (nitroprusside, nitroglycerin), n (%) | 15 (20%) | 26 (34%) | 0.05 | | ^b^ | |  |

a: Mann-Whitney test; b: Pearson's chi-square test; c: likelihood ratio test; d: Fisher's exact test.

CPB: cardiopulmonary bypass; IQR: interquartile range

**Additional file 1: Table S2.** Dobutamine use during surgery and in the first 7 days postoperatively according to dobutamine group.

| Variable | **Dobutamine-sparing** | **Dobutamine-**  **to-all** | p-value | | | |  |
| --- | --- | --- | --- | --- | --- | --- | --- |
|  | n=80 | n=80 |  |  |  |  |  |
| Dobutamine starting time, n (%) |  |  |  | | ^b^ | |  |
| In theatre, at CPB separation | 15 (19%) | 77 (96%) | X | |  | |  |
| In theatre, after CPB separation | 23 (29%) | 0 | X | |  | |  |
| In ICU | 13 (16%) | 0 | X | |  | |  |
| **Intraoperative** dobutamine  *(considering only patients who received dobutamine)* | | | |  | |  | |
| Duration of therapy (min), median (IQR) | 165 (110 - 290) | 195 (125 - 300) | 0.29 | | ^a^ | |  |
| Mean dose (mcg/kg/min), median (IQR) | 5.5 (5.0 - 9.8) | 5 (4.9 - 7.0) | 0.07 | | ^a^ | |  |
| Dobutamine use in the ICU |  |  | |  | |  | |
| Day 0 | 51/80 (64%) | 77/80 (96%) | 0.001 | | ^b^ | |  |
| Day 1 | 43/76 (57%) | 57/77 (74%) | 0.02 | | ^b^ | |  |
| Day 2 | 12/73 (16%) | 10/76 (13%) | 0.57 | | ^b^ | |  |
| Day 3 | 8/45 (18%) | 7/47 (15%) | 0.71 | | ^b^ | |  |
| Day 4 | 4/37 (11%) | 3/29 (10%) | 0.95 | | ^b^ | |  |
| Day 5 | 3/27 (11%) | 1/16 (6.3%) | 0.99 | | ^d^ | |  |
| Day 6 | 3/15 (20%) | 1/5 (20%) | 0.99 | | ^d^ | |  |
| Day 7 | 3/7 (43%) | 1/2 (50%) | 0.99 | | ^d^ | |  |
| Duration of inotropic therapy (min), median (IQR)  *(considering only patients who received dobutamine)* | | | | | | | |
| Day 0 | 840 (480 - 960) | 840 (600 - 960) | 0.63 | | ^a^ | |  |
| Day 1 | 840 (600 - 1440) | 720 (540 - 1080) | 0.27 | | ^a^ | |  |
| Day 2 | 1440 (1080 - 1440) | 1440 (600 - 1440) | 0.84 | | ^a^ | |  |
| Day 3 | 1350 (930 - 1440) | 1200 (450 - 1440) | 0.17 | | ^a^ | |  |
| Day 4 | 1440 (900 - 1440) | 660 (630 - 1050) | 0.72 | | ^a^ | |  |
| Day 5 | 1440** | 1440* | - | |  | |  |
| Day 6 | 1440** | 1440* | - | |  | |  |
| Day 7 | 1440** | 1440* | - | |  | |  |
| Mean dose (mcg/kg/min), median (IQR)  (considering only patients who received dobutamine) | | | | | | | |
| Day 0 | 5.2 (3.3 - 9.4) | 5.1 (4.1 - 7.1) | 0.57 | | ^a^ | |  |
| Day 1 | 3.9 (2.9 - 6.4) | 3.2 (2.6 - 5.2) | 0.48 | | ^a^ | |  |
| Day 2 | 4.9 (1.9 - 11.5) | 5.2 (2.7 - 7.6) | 0.70 | | ^a^ | |  |
| Day 3 | 7.9 (4.9 - 9.9) | 5.0 (2.4 - 5.6) | 0.63 | | ^a^ | |  |
| Day 4 | 5.7 (2.8 - 8.1) | 5.0 (4.1 - 12.4) | 0.56 | | ^a^ | |  |
| Day 5 | 6.4 (4.2 - 7.1) | 19.8* | - | |  | |  |
| Day 6 | 6.0 (4.0 - 6.9) | 19.8* | - | |  | |  |
| Day 7 | 6.0 (4.0 - 6.9) | 19.8* | - | |  | |  |

* One patient ** Three patients with same value

a: Mann-Whitney test; b: Pearson's chi-square test; c: likelihood ratio test; d: Fisher's exact test.

CPB: cardiopulmonary bypass; ICU: intensive care unit; IQR: interquartile range

**Additional file 1: Table S3.** Primary outcome subanalysis according to preoperative beta-blockers therapy

*Primary outcome was a composite of 30-day mortality and major cardiovascular complications (ventricular or supraventricular arrhythmias, acute myocardial infarction, stroke or transient ischemic attack, low-output syndrome and cardiogenic shock)*

| Variable | **Dobutamine-sparing**  *(n=80)* | **Dobutamine-**  **to-all**  *(n=80)* | p for interaction |
| --- | --- | --- | --- |
| Primary outcome In the 68 patients on preoperative beta-blockers, n (%) | 8/36 (22%) | 13/32 (41%) |  |
| Primary outcome In the 92 patients **not** on preoperative beta-blockers, n (%) | 17/44 (39%) | 14/48 (29%) |  |
|  |  |  | 0.063 |

**Additional file 1: Figure S 1**. Dobutamine dosage over time in the two groups

**Additional file 1: Figure S 2**. Mechanical ventilation for the first 7 days postoperatively according to dobutamine group


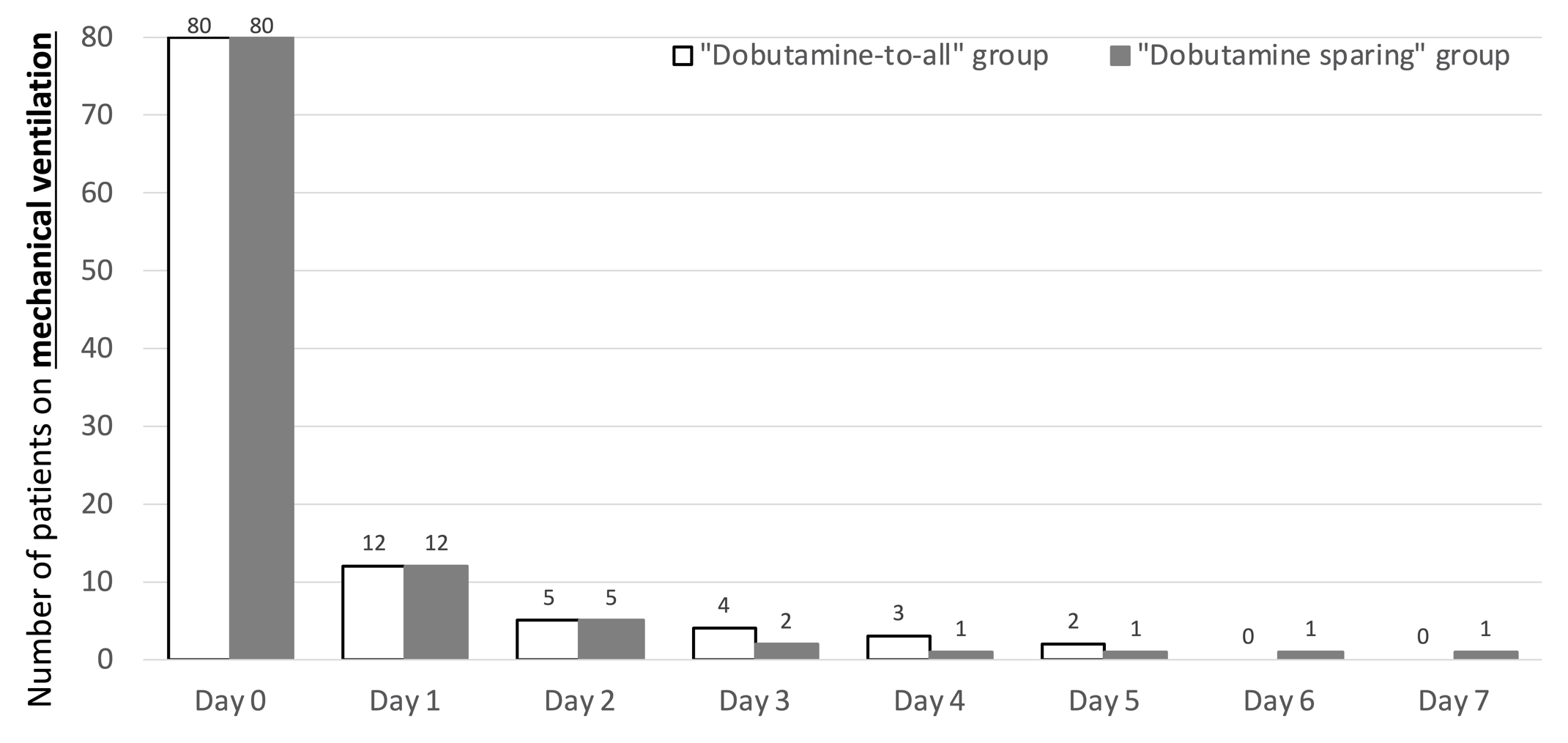


**Additional file 1: Figure S 3**. SOFA score (mean and standard deviation) the first 7 days postoperatively according to dobutamine group

**Additional file 1: Figure S 4**. Hemodynamic variables (mean and standard deviation) during the first 7 days postoperatively according to dobutamine group


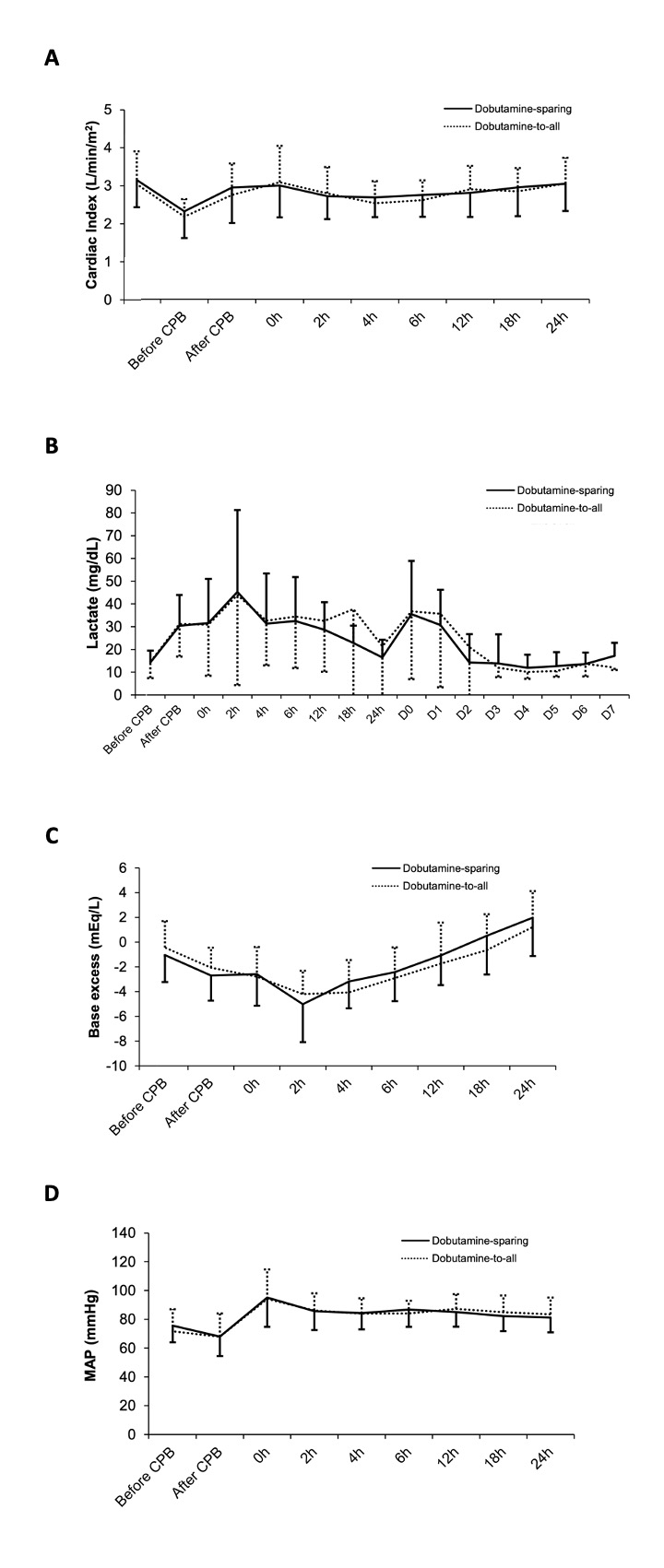


Panel A: Cardiac Index in the first 7 postoperative days

Panel B: Lactate in the first 7 postoperative days

Panel C: Base Excess in the first 7 postoperative days

Panel D: MAP in the first 7 postoperative days

**Additional file 1: Figure S 5**. Creatinine during the first 7 days postoperatively according to dobutamine group

**Additional file 1: Figure S 6**. Hemoglobin during the first 24 hours according to dobutamine group


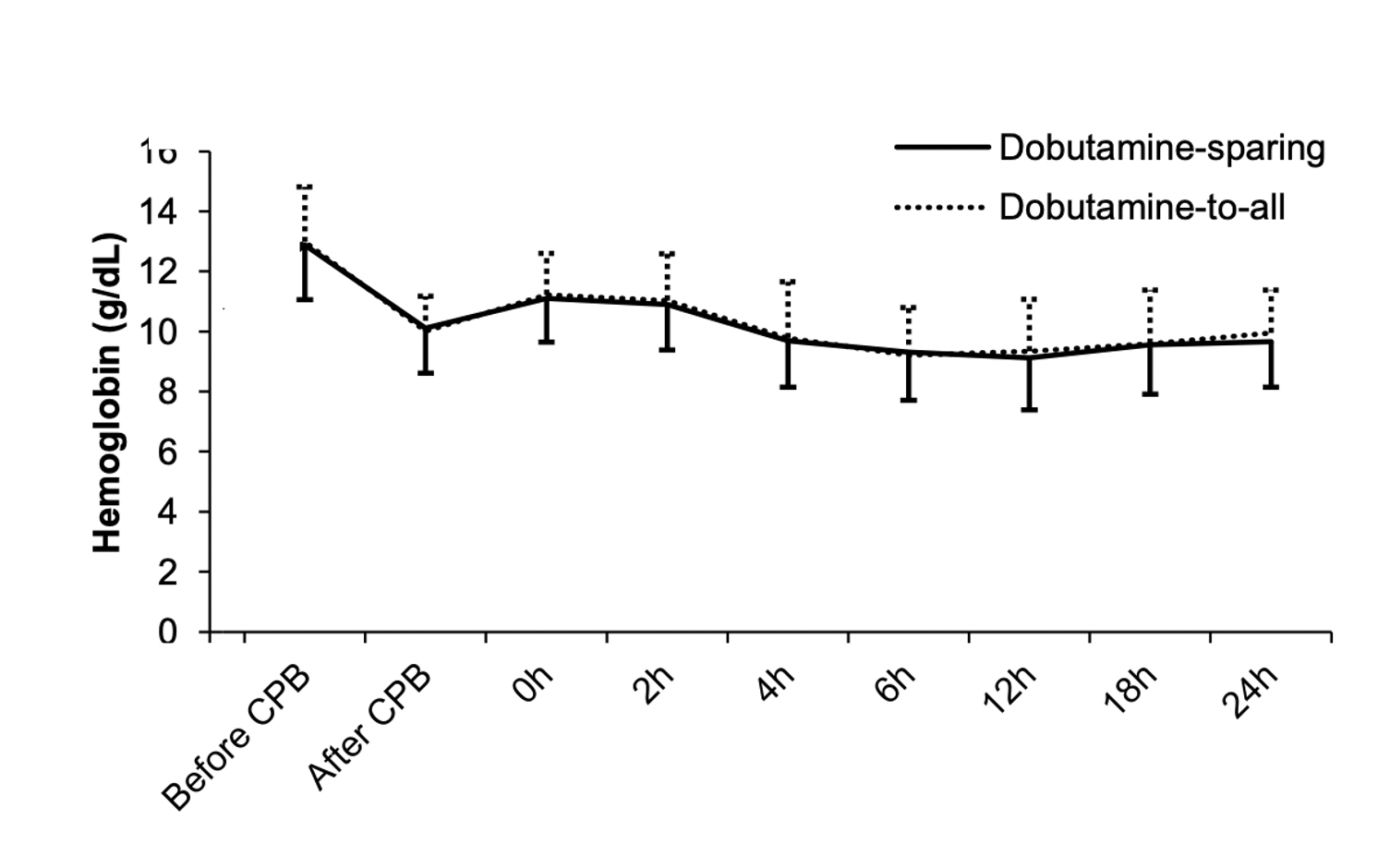

Supplement: Supplementary file 1 — Additional file 1: Material S1. Institutional Protocol for surgical and anesthetic management in cardiac surgery. Material S2. Comorbidities and outcomes definitions. Table S1. Intraoperative characteristics of patients according to dobutamine group. Table S2. Dobutamine use during surgery and in the first 7 days postoperatively according to dobutamine group. Table S3. Primary outcome subanalysis according to preoperative beta-blockers therapy. Figure S1. Dobutamine dosage over time in the two group. Figure S2. Mechanical ventilation for the first 7 days postoperatively according to dobutamine group. Figure S3. SOFA score (mean and standard deviation) the first 7 days postoperatively according to dobutamine group. Figure S4. Hemodynamic variables (mean and standard deviation) during the first 7 days postoperatively according to dobutamine group. Figure S5. Creatinine during the first 7 days postoperatively according to dobutamine group. Figure S6. Hemoglobin during the first 24 hours according to dobutamine group. [file 13613_2021_808_MOESM1_ESM.docx]
